# N-Containing Hybrid Composites Coatings for Enhanced Fire-Retardant Properties of Cotton Fabric Using One-Pot Sol–Gel Process

**DOI:** 10.3390/polym15020258

**Published:** 2023-01-04

**Authors:** Laila Khan, Jong Seop Kim, Seok-Hwan Huh, Bon Heun Koo

**Affiliations:** 1 Department of Materials Convergence and System Engineering, Changwon National University, Changwon 51140, Gyeongsangnam-do, Republic of Korea; 2 Department of Material Science and Engineering, Changwon National University, Changwon 51140, Gyeongsangnam-do, Republic of Korea; 3 Department of Mechatronics Convergence Engineering, Changwon National University, Changwon 51140, Gyeongsangnam-do, Republic of Korea

**Keywords:** microstructure, sol–gel, thermal properties, FTIR, UL–94, fire retardant, TGA

## Abstract

In this report, a unique methodology/process steps were followed using Sol–gel-based concept to deposit thin flame-retardant coatings on cotton fabric. Surface microstructure and compositional analysis of the coated cotton were carried out using scanning electronic microscope (SEM), which explored significant coverage of the fabric. The obtained samples were further analyzed through rupturing mechanism test and color check. Compositional investigation of the coated samples was carried through Attenuated total reflection Fourier transform infrared (ATR–FTIR) and energy-dispersive X-rays spectroscopy (EDS) analysis. Thermal analyses were carried out through Thermogravimetric analysis (TGA) and Vertical flame tests (VFT), which suggested higher resistance of the coatings obtained for 5 h and zero heat-treatment time on the cotton fabric. A 28.86% char residue was obtained for the same sample (ET–5h–RT) coupled with higher degradation temperature and excellent combustion properties.

## 1. Introduction

Textile fabrics have been known to human beings for a long time and have been utilized for traditional purposes, as well as in advanced technology ranging from indoor interior application to structural and functional applications [1,2,3]. However, their intrinsic high flammability and ignitibility contribute to their decreasing application and functional failures due to extreme fire hazards caused by these textile fabrics [4,5]. The available commercial solutions in the form of Pyrovatex CP^®^ or Proban^®^ for various types of textile fabrics provide a satisfactory recipe for a cost-effective process and availability [6]. However, the modern national and international laws/regulations regarding the textile flame-retardants have become more severe and inflexible in order to avoid toxicological issues related to these synthetic chemicals [7]. In this regard, the most significant are halogen-based compounds that can produce toxic corrosive gases during the burning process [8]. Phosphorus-based compositions such as tetrakis hydroxymethyl phosphonium chloride (THPC) and N-methylol dimethyl phosphono-propionamide (MDPA), which can release formaldehyde, were highly discouraged. To cope with these issues, a broad variety of research studies have been pursued, which mainly focused on surface modification techniques that can use chemical flame-retardant agents to impart flame retardancy to the fabric. The major surface treatment and modification techniques include layer-by-layer techniques [9,10,11,12,13,14,15,16,17], the application of reactive monomeric or polymeric species [18,19,20,21], phosphorous-containing biomolecules such as DNA or casein [20,22,23], UV-curable [24,25] or plasma-curable systems [26,27,28,29,30,31] and coatings based on Sol–gel techniques [32,33,34,35,36,37,38]. For several reasons, interest in the Sol–gel processes in the textile field has grown in a remarkable manner. The Sol–gel approach has been recently used for several new functional properties and applications, such as antimicrobial or UV radiation protection [39,40,41,42,43,44], dye fastness, anti-wrinkle finishing, super-hydrophobicity, and immobilization of biomolecules, among other uses. In particular, it has been clearly demonstrated that Sol–gel-derived hybrid architectures are capable of protecting the polymer surface and acting as a thermal insulator, thus improving the flame retardancy of the treated substrates [45,46,47]. More specifically, referring to cellulosic substrates, these inorganic architectures absorb the heat from the surrounding area, and thus are able to protect the polymer substrate by creating a physical barrier to oxygen and heat transfer. In doing so, they hinder the formation of volatile species that fuel further degradation and, at the same time, favor the formation of a carbonaceous structure (char) [48,49]. One stage included simply doping flame-retardants physically into silica sol during the Sol–gel process, in which the treated fabrics possessed poor washing durability. Meanwhile, increasing attention has focused on the use of an alkoxysilane precursor bearing a flame-retardant atom such as phosphate, boron, and nitrogen to form chemically hybrid silica sol via the Sol–gel method [50,51]. Additionally, Alongi et al. [36] successfully used diethyl phosphatoethyl triethoxysi-lane (DPTES) as a monomer to synthesize hybrid phosphorus–silicon organic–inorganic coatings to enhance cotton flame-retardancy. This strategy sounds like a promising way to improve the washing durability of sol coating for the incorporation of hybrid element into the sol matrix via chemical bonding. However, it is difficult to obtain these precursors carrying phosphorus, boron, or nitrogen. It is reported that Yang Z.Y synthesized a novel flame-retardant precursor containing phosphorus and nitrogen using diphenylphosphinic chloride and (3–aminopropyl) trimethoxysilane as reactants for flame retardancy of cotton. It is well known that the inorganic borax and borate are not washing durable for finishing, and the drawback of organic boron compounds as flame retardants relates to their poor hydrolytic stability owing to the electron deficiency of boron atoms. Consequently, efforts have been made to replace boron composite flame retardants by introducing new elements such as nitrogen or silicon into the same structure system, with improved flame retardancy and washing durability. In the present work, a hybrid silica sol in combination with melamine (N–precursor; 99%) was obtained through the Sol–gel method. The precursor tetraethoxysilane (TEOS;99.0%) reacted with N-species, which played the role of flame retardant and catalyst via the cohydrolysis and condensation reaction at a relatively low temperature. Thus, a homogeneous hybrid sol system was obtained, in which the trigonal NO_3_ units would be incorporated into the siloxane network via Si–O–N bridges [37]. The coated samples were characterized through various techniques in order to assess the thermal stability and characterization of the microstructure of the coatings.

## 2. Experimental Section

### 2.1. Materials

Original Cotton fabric (100%, 180 g m^−2^) was used as an experimental substrate material. Before use, the fabric was divided carefully into sampling pieces (height/width ~ 230 mm/120 mm), cleaned, and dried. Ethanol and HCl were purchased from Samchun Chemical Co., Ltd., Seoul, Republic of Korea. Melamine and Tetraethoxysilane (TEOS) were purchased from Sigma Aldrich, Burlington, MA, United States. The DI water used in the experiment was of 18.6 Ω∙cm.

### 2.2. Preparation of Sol

The sol was prepared using the chemical with the given rations of TEOS: ETHANOL: DI = 1:2:4. After complete mixing of these chemicals, 10 mL of HCl (37%) was added to the mixture and stirred to room temperature for 4 h until a clear transparent sol was formed. Meanwhile 10% melamine solution was prepared using Ethanol. The 10% melamine solution was mixed with the sol during the stirring process. The sol was then put in a 500 mL beaker for cotton impregnation.

### 2.3. Cotton Treatment

Ten pieces of cotton fabric samples (230 mm × 120 mm) numbered in sequence were used for the treatment. For each experimental condition, two samples were used. Sample 1 and 2 were control cotton fabric. Samples 3 and 4 (ET–0.5h–15) were treated in the melamine-based solution for 0.5 h under the condition of drying at 90 °C for 15 h and curing at 165 °C for 1.0 min. Samples 5 and 6 (ET–2h–15), were treated in the melamine-based solution for 2 h under the condition of drying at 90 °C for 15 h and curing at 165 °C for 1.0 min. Samples 7 and 8 (ET–14h–5), were treated in the melamine-based solution for 14 h under the condition of drying at 90 °C for 5h and curing at 165 °C for 1.0 min. Sample 9,10 (ET–5h–RT) were treated in the melamine-based solution for 5h under the condition of drying at RT and curing at 165 °C for 1.0 min.

### 2.4. Characterization Techniques

Using a low-voltage scanning electronic microscope (LV–SEM, Merlin compact, Zeiss, Oberkochen, Germany), the surface morphologies of the coated and the untreated fabric samples were examined. EDS (energy-dispersive X-rays spectroscopy) was used as a combined means of characterization along with LV–SEM to help examine the elemental composition of the coating surfaces (vide infra). For effective surface microscopy, the as-coated fabric samples were Pt sputtered for short period of 3 min, subject to a high vacuum to help transmit conduction to the fabric surface. The coated samples were then analyzed through attenuated total reflection Fourier transform infrared (ATR–FTIR) spectra (JASCO 6300, Easton, MD, United States), in the frequency range of 4000 to 400 cm^−1^. The spectra profiles were recorded after 32 scans at a resolution of 4 cm^−1^. The thermal stability of uncoated and coated fabrics was then analyzed in the nitrogen atmosphere (20 mL min^−1^) using Scinco Thermogravimetric Analyzer (N–1000/1500, SCINCO, Seoul, Republic of Korea) and in the temperature range of 50 to 600 °C, at a heating rate of 20 °C min^−1^. A quantity of each sample used for TGA count was taken ~ 8–10 mg. Macro-combustion tests such as the vertical flame test (VFT) was performed for each coated and the control sample, in conformity with documented ASTM D6413 standards. Accordingly, the samples were combusted in a lab-made vertical flammability box (300 mm/120 mm) using a Bunsen burner flame; the flame was applied for 10 s, exactly 20 mm below the fabric sample and the process was recorded using a high-speed optical camera.

## 3. Results and Discussion

### 3.1. Surface and Compositional Analysis

**Figure 1:**
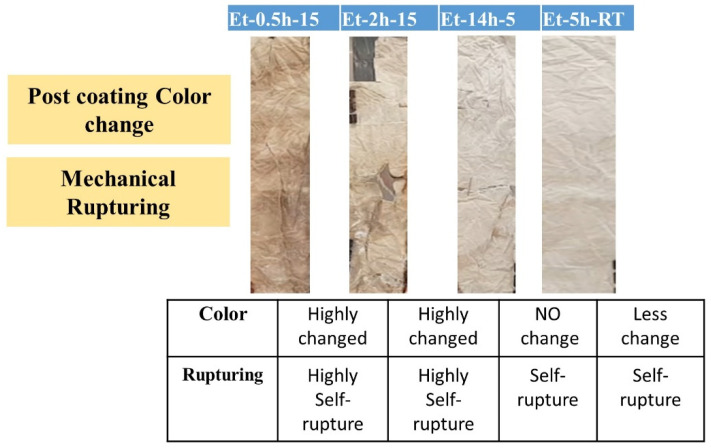
Typical images of the coated cotton fabric.

General images of the coated cotton processed under various conditions were taken and compared with the uncoated cotton fabric, as shown in Figure 1. The images were taken after completion of all steps, including coating, drying, curing, and then washing. Various conditions of the coating preparation and then post-coating conditions can be seen in Table 1. The typical images not only show color difference imparted to the cotton fabric during the coating process, but also the intensity and degree of self-rupturing of the cotton fabric due to the treatment process. It can be seen that the cotton heated for a long time, as shown in ET–0.5h–15 (15 h heating), ET–2h–15 (15 h heating) has a high degree of color difference and self-rupturing compared to the other samples. The inset table in Figure 1 shows the level of rupturing in all the coated fabric. Broken patches and parts caused by the self-rupturing can be easily seen in the images in ET–0.5h–15, ET–2h–15, suggesting the coated process has severely danged the cotton fabric to the extent that it could not afford shaking or transference from one place to other. Thus, in terms of initial practical observation, the condition is not feasible for the objective application of these coatings. In addition, it can be seen that as well as the self-rupturing, the colors of the cotton have been changed to a higher degree from initial un-coated fabric. It is believed that due to a longer duration of heating, the presence of acidic molecules was responsible for such adverse changes in the originality of the cotton fabric. As the heating time decreased from 15 h to 5 and 0 h, the cotton fabric regained its original color and ability to withstand self-rupturing. The heating process could cause the acidic and silica network to react in such a manner, possibly causing an exothermic reaction in the presence of a high-heat futile environment, and thus, it could damage the cellulosic network of the cotton fabric, as can be seen in the initial two samples. However, as the heating time has been reduced, the level of exothermic reaction and effects were reduced, and thus less color change and rupturing were observed.

**Figure 2:**
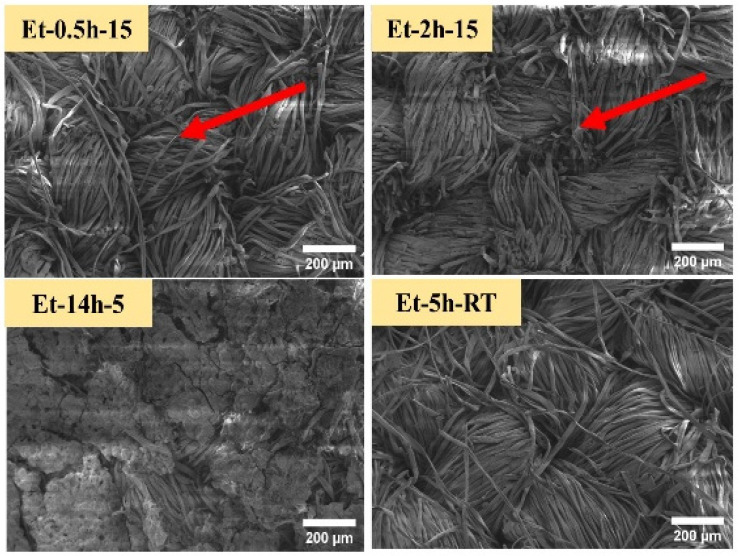
SEM iamges of the cotton fabric obtained under various conditions.

The coated cotton samples were than observed through low-voltage scanning electron microscopy, as shown in Figure 2. Since the cotton fabric is cellulosic in nature, which is a green molecule that contains a combination of oxygen and carbon molecules, and thus, the high voltage can damage these molecular networks. The low-voltage SEM was able to observe the micro-details of the coatings deposited on the cotton fabric at the individual fiber level. From the SEM images, it can be seen that similar to macro-images, as discussed earlier, the rupturing process penetrated to the singular fibers. A portion of fibers can be seen as broken and torn apart, as shown in ET–0.5h–15, ET–2h–15. In addition, it can that the coating deposition performed uniformly on all the individual fibers, and thus covered the entire fabric matrix. Further, it can be seen that the coating process could not change the original and compact nature of the cotton fabric, and thus, the design and pattern of the cotton fabric is still in place, as found in the control cotton fabric. Coating microstructure found on ET–14h–5, ET–5h–RT can be seen as different compared to the initial two conditions. In case of ET–14h–5, the surface is completely covered, and thus the individual fabric cannot be seen clearly, while in the case of ET–5h–RT, the surface is compact and has no broken portions or parts, as found earlier, confirming the lesser extent of rupturing. Further, the original pattern of the fabric was found to be intact and compact. Surface morphology and microstructure as observed in the SEM can be helpful to discuss the results obtained in the next experiments.

**Figure 3:**
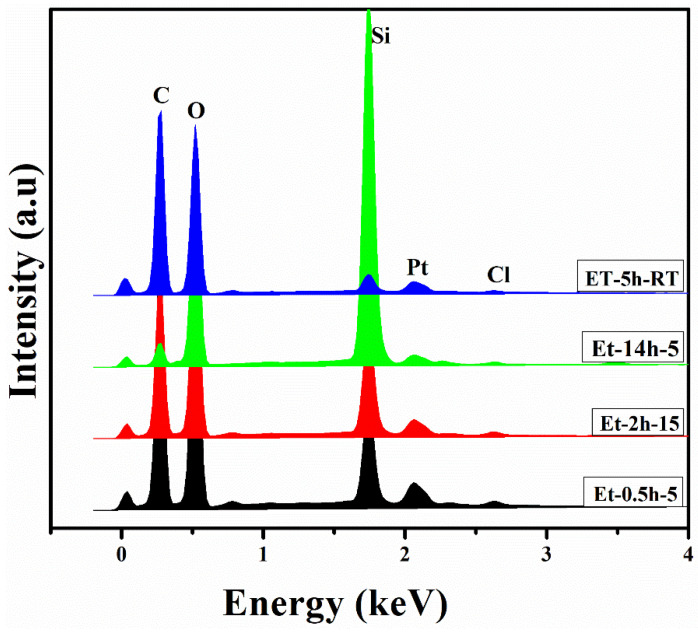
EDS profile of the coated fabrics.

To determine the compositional profile of the coatings deposited on the cotton fabric, EDS analysis was carried out and the elemental profiles for each coating were drawn as shown in Figure 3. It can be seen that C, O, Si, Pt, and Cl were obtained in the elemental profiles. C and O were obtained from the ethanol and cotton fabric, while the Si was obtained from the TEOS. Pt was obtained from the Pt sputtering, carried out before the SEM and EDS measurement to facilitate conductivity of the coated fabric. Si was obtained from the TEOS and Cl was obtained from the HCl. N was deposited in a lesser amount from the precursors and thus could not be found in the elemental profiles. The presence of an excessive amount of Si suggests the successful deposition of the Sol–gel to the individual fiber, and thus could help to protect the coated fabric against fire.

**Figure 4:**
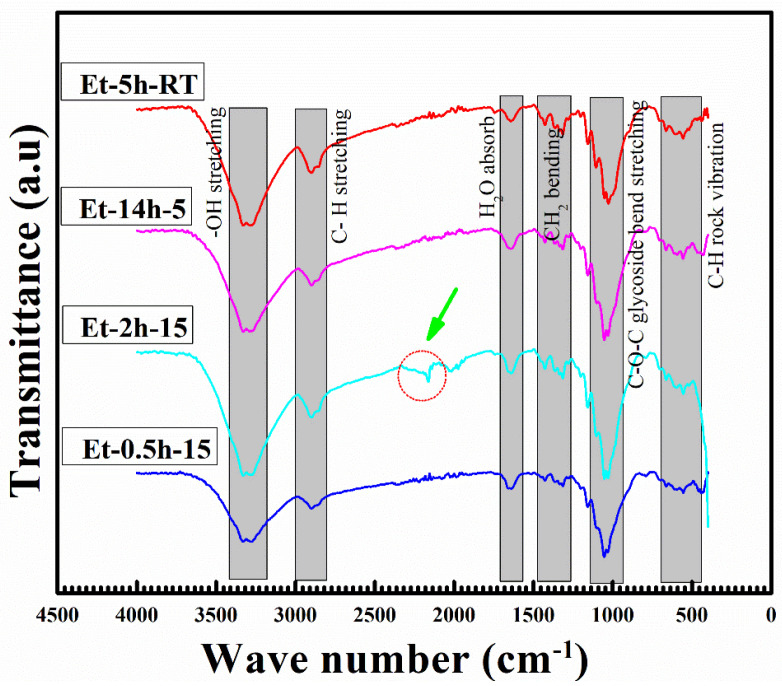
FTIR analysis profile of the coated cotton fabrics.

The FTIR analysis, which is used to explore the molecular composition of the coated species, can be seen in Figure 4. The obtained peaks in the FTIR profile can be attributed to various stretching and vibrational modes of the bonds between these species. Major stretching and vibrational modes were recorded as follows: OH stretching, CH stretching, H_2_O absorption, CH absorption, CH_2_ bending, C–O–C glycoside bend stretching and C–H rock vibration. The OH and CH stretching were obtained at above 2500 cm^−1^ wave number, suggesting lower energies for these resonations. It is believed that these vibrations have been caused by the C–H and O–H present in the ethanol and cotton fabric. Further, it can be seen that width of these two peaks has been altered significantly with coating conditions, suggesting OH is removed by a longer heating process. It is important to note that the peak shift and modification obtained at 1200 and 1135 cm^−1^ can be attributed to the stretching of the –Si–O– cellulose and –Si–O–Si– bonds, which suggests the successful deposition of the coating layers.

### 3.2. Thermal Analysis

**Figure 5:**
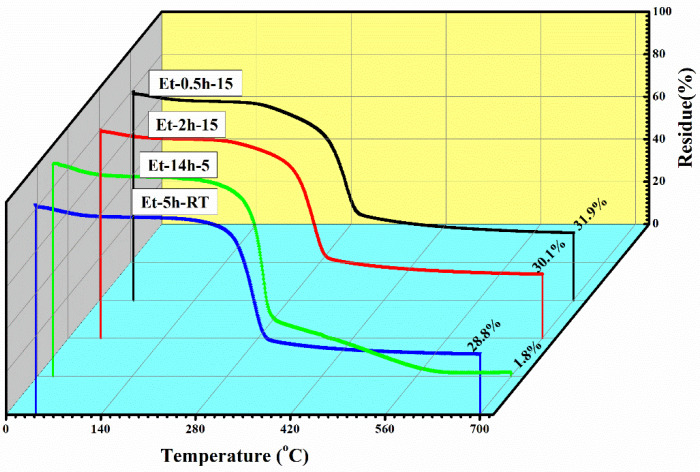
Thermogravimetric analysis curves of the coated fabric obtained under various conditions.

**Figure 6:**
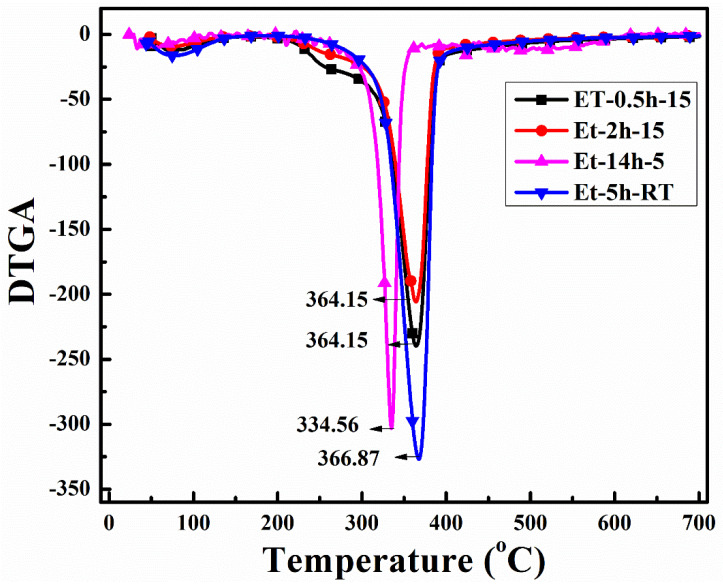
DTGA analysis curves of the coated fabrics obtained under various conditions.

**Figure 7:**
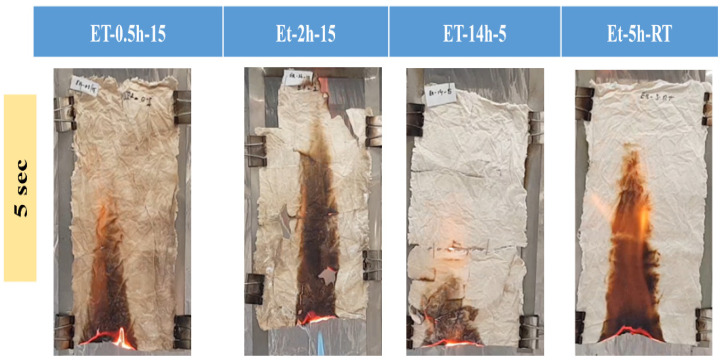
Images of the coated samples obtained after 5 s of flame initiation.

**Figure 8:**
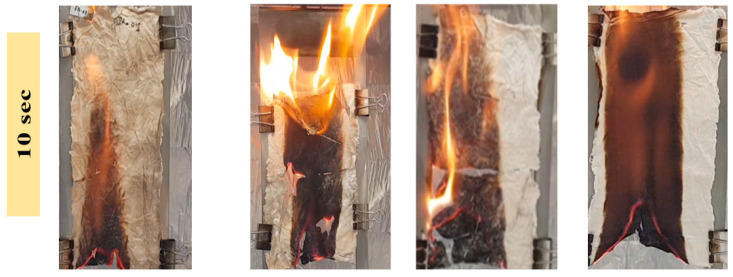
Images of the coated samples obtained after 10 s of flame initiation.

**Figure 9:**
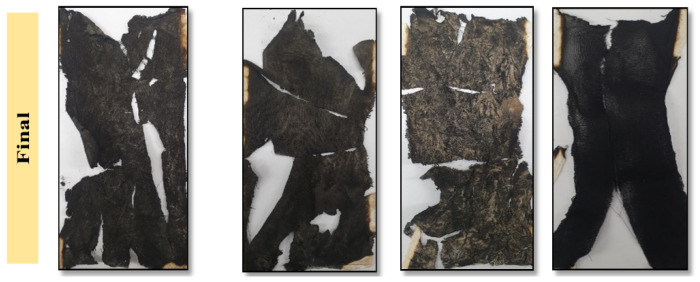
Final images of the coated sample completely converted to char as a result of VFT test.

To observe the thermal protection efficiency of the coated cotton, TGA analysis was carried out for all the coated samples. The TGA was carried out from 25 °C to 700 to 20 °/min, as shown in Figure 5. The TGA results were drawn as weight loss (%) on the *y*-axis and temperature increase on the *x*–axis. It can be seen that, apparently, 30% of residue was obtained for all the coatings. The higher residue obtained for ET–0.5h–15 and ET–2h–15 was because they all the low-temperature degradation components were lost during the longer heating process, and thus sustained the temperature during TGA. The results were further confirmed by the T_5%_ values ~ 110.70 °C for ET–2h–15, which were higher than all other samples, as mentioned in Table 1. Various parameters obtained from the TGA can be seen in Table 1. The DTGA profile was obtained to determine the highest changes in the degradation and temperature where higher changes occurred, as shown in Figure 6. The highest temperature was recorded as 366.87 °C, while the lowest temperature was found to be ~ 334.56 °C, as mentioned in Table 1. The highest temperature suggests higher resistance to the flame provided by the coated materials deposited on the cotton fabric. To further analyze the thermal properties of the coated samples, a vertical flame test was carried out and the images were taken at intervals of 5 s, 10 s, and final burn in order to observe the flame spread properties. A VFT test was carried out in a lab-made chamber according to the ASTM D 6413 Standard Test Method for Flame Resistance of Textiles procedure. The coated samples with the specific dimensions stated earlier were exposed to the methane flame at 10 cm distance for 10 s. After the removal of flame at 10 s, images of burning samples and other parameters were calculated accordingly. It can be seen that at 5 s, as in Figure 7, the ET–5h–5 and ET–14h–RT samples were significantly resistant to the flame compared to other samples. However, no sample resisted the flame to the level of extinguishing. The images taken at 10 s can be seen in Figure 8, which shows that the flame spread has almost covered the sample area in vertical and horizontal directions. Compared to other samples, the ET–5h–5 and ET–14h–RT partially blocked the flame spread in the mid-region section, and thus, the obtained flame intensity was minimal compared to other samples. The final images obtained from the VFT analysis can be seen in Figure 9. The final images are important to observe because their mechanical stability prohibit the transfer of flame to other areas through disintegration, and the large amount of residue supports the efficient role of the coatings.

**Figure 10:**
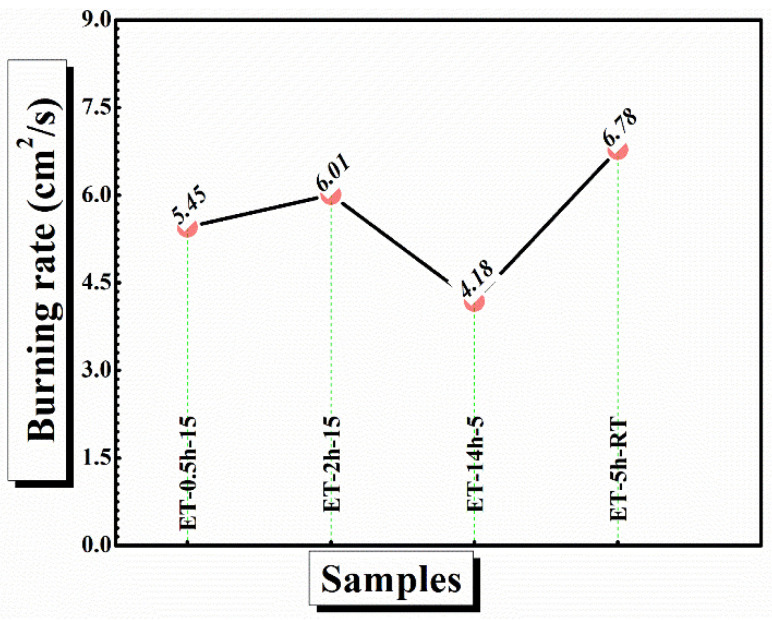
Burning rate of the coated cotton obtained from VFT analysis.

In case of ET–0.5h–15 and ET–2h–15, the char has been completely broken into parts and could not withstand alone, suggesting strain in the cotton matrix and conversion of the maximum portion of the char into ash. In comparison, the ET–5h–5 and ET–14h–RT can be seen as mechanically stable and thus could play a role to block the transfer of flame from one place to other, which could not be the case if the cotton broke into pieces and was thrown into a new area of flame-less zone [48]. Finally, the burning rate of the coatings was calculated from the VFT experiment as shown in Figure 10. The lowest burning rate was obtained as 4.1 cm^2^/s for ET–5h–5, which is quite a bit lower than the values obtained for other samples.

## 4. Conclusions

Sol–gel coatings were successfully deposited onto the cotton fabric for fire-retardant applications. It was found that under a longer heat-treatment time of 15 h, the original color and mechanical properties of the cotton were severely altered. However, for shorter treatment times of 5 h and 0 h, the color and mechanical properties of the cotton were found to be immune to the coating process. The highest degradation temperature was obtained for ET-5h-RT ~ 383.02 °C, with significant resistance to the degradation process in the initial stages. Likewise, the peak degradation temperature was at its highest ~ 366.87 °C for ET–5h–RT coupled with significant char residue ~ 28.86%. The results were further confirmed by the flame spread and burning rate profile, with the lowest flame spread obtained for ET–5h–RT.

## Figures and Tables

**Table 1:** Thermogravimetric analysis parameters of the coated samples.

#	T_5%_	T_on_	R_on_	T_off_	R_off_	T_peak1_	T_peak2_	Total Decomposition %
**ET–** **0.5h–** **15**	102.02	245.16	93.26	383.02	38.61	254.90	364.15	31.92
**ET–** **2h–** **15**	110.70	315.22	93.30	380.75	35.13	252.98	364.15	30.10
**ET–** **14h–** **5**	92.20	312.96	93.15	338.08	27.33	–	334.56	1.80
**ET–** **5h–** **RT**	99.75	328.26	92.27	383.02	33.11	–	366.87	28.86

## Data Availability

Not applicable.
